# Supplementary material for: The interactome of intact mitochondria by cross-linking mass spectrometry provides evidence for coexisting respiratory supercomplexes
Source: Mol Cell Proteomics. 2017 Dec 8;17(2):216–32. doi: 10.1074/mcp.RA117.000470 (PMC5795388; doi:10.1074/mcp.RA117.000470)
Supplement: Supplemental Data [file supp_RA117.000470_133922_0_supp_23978_fzffwf.zip › spectra_annotation/mito_spectra_annotation/293-1-6-1-22-1.pdf]

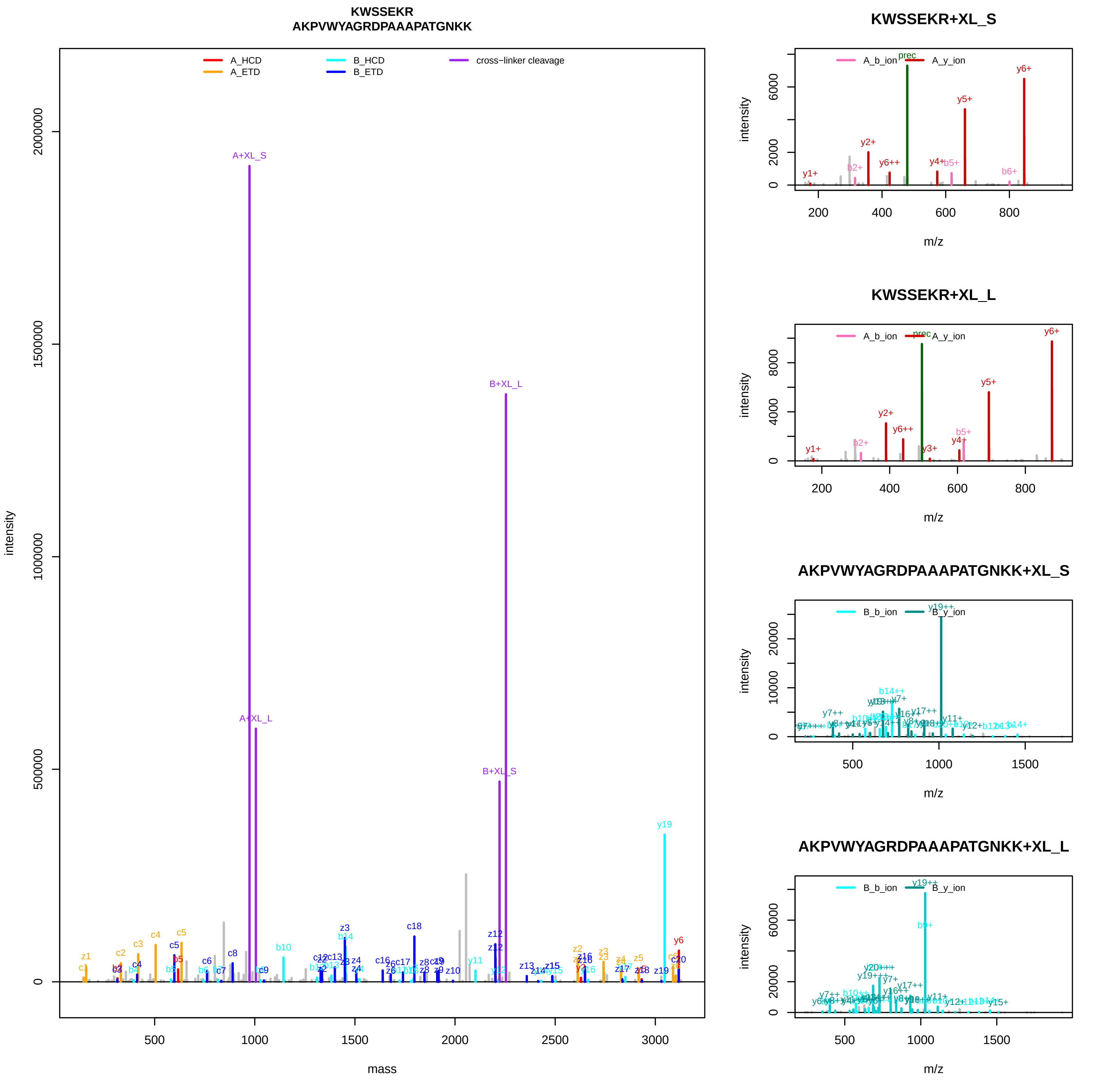

KWSSEKR
AKPVWYAGRDPAAAPATGNKK
A_HCD
A_ETD
B_HCD
B_ETD
cross−linker cleavage
A+XL_S
A+XL_L
B+XL_L
B+XL_S
y19
c18
z3
b14
z12
b10
y6
intensity
mass
0
500000
1000000
1500000
2000000
500
1000
1500
2000
2500
3000
KWSSEKR+XL_S
A_b_ion
A_y_ion
prec
y1+
b2+
y2+
y6++
y4+
b5+
y5+
b6+
y6+
m/z
KWSSEKR+XL_L
y3+
AKPVWYAGRDPAAAPATGNKK+XL_S
B_b_ion
B_y_ion
y19++
b14++
AKPVWYAGRDPAAAPATGNKK+XL_L
b9+
